# Frontiers Research Topic: Radiation-Induced Effects and the Immune System

**DOI:** 10.3389/fonc.2013.00055

**Published:** 2013-03-25

**Authors:** Gabriele Multhoff, Franz Rödel, Alan G. Pockley, Udo S. Gaipl

**Affiliations:** ^1^Radiation Oncology, Klinikum Rechts der Isar, Technische Universität MünchenMunich, Germany; ^2^Radiation Therapy and Oncology, University Hospital FrankfurtFrankfurt, Germany; ^3^John van Geest Cancer Research CentreNottingham, UK; ^4^Radiation Oncology, University Hospital ErlangenErlangen, Germany

The development of novel, more precise instruments for the delivery of ionizing radiations, the accretive utilization of protons and heavy ions, and the emphasis on hypofractionated irradiation schemes have resulted in major improvements in locoregional tumor control. However, radiation therapy alone is still insufficient to cure locally advanced metastatic tumors. The heterogeneity of neoplasms and their microenvironment also affect the radiosensitivity of cancers. Thus, there is an urgent clinical need for innovative therapeutic concepts that may be translated into novel radio(chemo)therapeutic regimens. In order to develop new therapeutic strategies, a profound knowledge of tumors and their microenvironment is essential.

In this e-book, the authors deal with various aspects of interrelationship between neoplasms and their microenvironments, including how high and low dose irradiation affects the immune system, the effects of targeted and non-targeted photon, and particle irradiation on tumor and normal tissues, as well as the impact of epigenetics on irradiation therapy. Furthermore, the authors report on the effects of irradiation on inflammatory pathways and on the microcirculation, two aspects that are highly relevant for the delivery of macromolecules and other therapeutics to irradiated tumors. A major focus of this e-book is to elucidate immunological facets of ionizing irradiation. It is indeed becoming clear that high- and low-dose irradiation elicit distinct immunological effects. Furthermore, the mode of irradiation-induced cell death (e.g., apoptosis, necrosis) has a major impact on anticancer immune responses. Overall, the e-book aims at review the current knowledge on the role of ionizing irradiation in antitumor immunity. This knowledge is instrumental for the development of innovative therapeutic modalities involving the combination of radio(chemo)therapy and immunotherapy in patients with locally advanced tumors.

